# T‐cell Immunoglobulin and ITIM Domain Contributes to CD8^+^ T‐cell Immunosenescence

**DOI:** 10.1111/acel.12716

**Published:** 2018-01-19

**Authors:** Yangzi Song, Beibei Wang, Rui Song, Yu Hao, Di Wang, Yuxin Li, Yu Jiang, Ling Xu, Yaluan Ma, Hong Zheng, Yaxian Kong, Hui Zeng

**Affiliations:** ^1^ Beijing Key Laboratory of Emerging Infectious Diseases Institute of Infectious Diseases Beijing Ditan Hospital Capital Medical University Beijing China; ^2^ Beijing Key Laboratory of Emerging Infectious Diseases The National Clinical Key Department of Infectious Disease Beijing Ditan Hospital Capital Medical University Beijing China; ^3^ Lab for Molecular Biology Institute of Basic Theory on Chinese Medicine China Academy of Chinese Medical Sciences Beijing China; ^4^ Penn State Hershey Cancer Institute Penn State University College of Medicine Hershey PA USA

**Keywords:** aging, CD8^+^ T cells, co‐inhibitory receptors, T‐cell immunosenescence, TIGIT

## Abstract

Aging is associated with immune dysfunction, especially T‐cell defects, which result in increased susceptibility to various diseases. Previous studies showed that T cells from aged mice express multiple inhibitory receptors, providing evidence of the relationship between T‐cell exhaustion and T‐cell senescence. In this study, we showed that T‐cell immunoglobulin and immunoreceptor tyrosine‐based inhibitory motif (ITIM) domain (TIGIT), a novel co‐inhibitory receptor, was upregulated in CD8^+^ T cells of elderly adults. Aged TIGIT ^+^ CD8^+^ T cells expressed high levels of other inhibitory receptors including PD‐1 and exhibited features of exhaustion such as downregulation of the key costimulatory receptor CD28, representative intrinsic transcriptional regulation, low production of cytokines, and high susceptibility to apoptosis. Importantly, their functional defects associated with aging were reversed by TIGIT knockdown. CD226 downregulation on aged TIGIT ^+^ CD8^+^ T cells is likely involved in TIGIT‐mediated negative immune suppression. Collectively, our findings indicated that TIGIT acts as a critical immune regulator during aging, providing a strong rationale for targeting TIGIT to improve dysfunction related to immune system aging.

## 1 INTRODUCTION

Immunosenescence is the age‐associated dysregulation of the immune system (Miller, 1996; Shaw, Goldstein, & Montgomery, 2013). It involves a gradual deterioration of cellular and humoral immunity during aging (Linton & Dorshkind, 2004; Nikolich‐Zugich, 2008). In addition, an inadequate increase in inflammation can be detrimental in elderly individuals (Shaw et al., 2013). Immunosenescence is of high clinical relevance, as it contributes to multiple age‐related comorbidities, including malignancies, infectious diseases, autoimmune diseases, and degenerative diseases (Gavazzi & Krause, 2002; Nikolich‐Zugich, 2008). T cells are important components of the immune system. Age‐associated T‐cell dysfunction is important for the development of immunosenescence (Chou & Effros, 2013; Moro‐Garcia, Alonso‐Arias, & Lopez‐Larrea, 2012).

T‐cell senescence is different from T‐cell exhaustion, a hyporesponsiveness associated with chronic infections and cancer. Exhausted T cells are derived from activated T cells that progressively lose function because of persistent antigen stimulation, whereas senescence is cell cycle arrest due to aging (Akbar & Henson, 2011; Crespo, Sun, Welling, Tian, & Zou, 2013). However, emerging evidence indicates that T‐cell senescence shares several key features with exhaustion. The upregulation of multiple co‐inhibitory receptors is not only a hallmark, but also an important mechanism involved in the development of T‐cell exhaustion (Wherry, 2011; Wherry & Kurachi, 2015). Consistently, certain co‐inhibitory receptors such as programmed cell death protein 1 (PD‐1), T‐cell immunoglobulin domain and mucin domain 3 (TIM‐3), lymphocyte activation gene 3 (LAG‐3), and cytotoxic T lymphocyte‐associated antigen‐4 (CTLA‐4) are upregulated on T cells from aged mice, and blockade of PD‐1 partially restores the functional defect of T cells derived from these mice (Channappanavar, Twardy, Krishna, & Suvas, 2009; Decman et al., 2012; Lee et al., 2016; Shimada, Hayashi, Nagasaka, Ohno‐Iwashita, & Inomata, 2009). This finding indicates a pivotal role of T‐cell inhibitory receptors in immunosenescence. However, whether the suppressive pathways contribute to immunosenescence in humans has not been addressed. In addition, the effect of newly identified co‐inhibitory receptors in this process needs to be investigated.

T‐cell immunoglobulin and immunoreceptor tyrosine‐based inhibitory motif (ITIM) domain (TIGIT) is a recently identified co‐inhibitory receptor that is expressed on activated T cells, regulatory T cells, and NK cells (Boles et al., 2009; Stanietsky et al., 2009; Stengel et al., 2012; Yu et al., 2009). Similar to CTLA‐4 and CD28, TIGIT competes with its costimulatory counterpart CD226 for the same ligands (CD155 and CD112) and mediates immune suppression in tumors and chronic infections (Chew et al., 2016; Johnston et al., 2014; Kong et al., 2016). Here, we investigated the role of TIGIT in human immunosenescence using blood samples from healthy adults. The present results demonstrate that TIGIT upregulation is an important process associated with senescent CD8^+^ T cells.

## 2 RESULTS

### 2.1 Age‐related TIGIT upregulation in CD8^+^ T cells

T cells from aged mice are characterized by high levels of the co‐inhibitory molecules PD‐1, TIM‐3, CTLA‐4, and LAG‐3. To determine whether these exhaustion markers are also associated with T‐cell aging in humans, we performed flow cytometric analysis of a number of co‐inhibitory surface molecules on T cells from 164 healthy adults (Table 1). Unlike the pattern in T cells from mice, comparable levels of PD‐1 and TIM‐3 and slight elevations of CTLA‐4 and LAG‐3 were observed in T cells from older adults (61–80 years old) compared with those in young (21–30 and 31–40 years old) or middle‐aged adults (41–50 and 51–60 years; Figure S1).

**Table 1:** Characteristics of cohort

Parameters	Total (*n* = 164)	20–30 (*n* = 37)	31–40 (*n* = 37)	41–50 (*n* = 27)	51–60 (*n* = 28)	61–80 (*n* = 35)	*p* Value
Gender							
Male	84	18	24	14	11	17	.3362
Female	80	19	13	13	17	18	
Age, years							
Median	45	28	36	45	54	70	<.0001
IQR	32.25–61	25.75–29	33.25–37	42.5–48	52.75–59.25	65–73	

A total of 164 healthy volunteers were recruited, including 84 males and 80 females. Their median age was 45, and 27–37 adults were in every group. The chi‐square test demonstrated that the gender was balanced among all the groups (*p* = .3362). Age was described by median and interquartile range (IQR) and analyzed using Kruskal–Wallis test.

**Figure 1:**
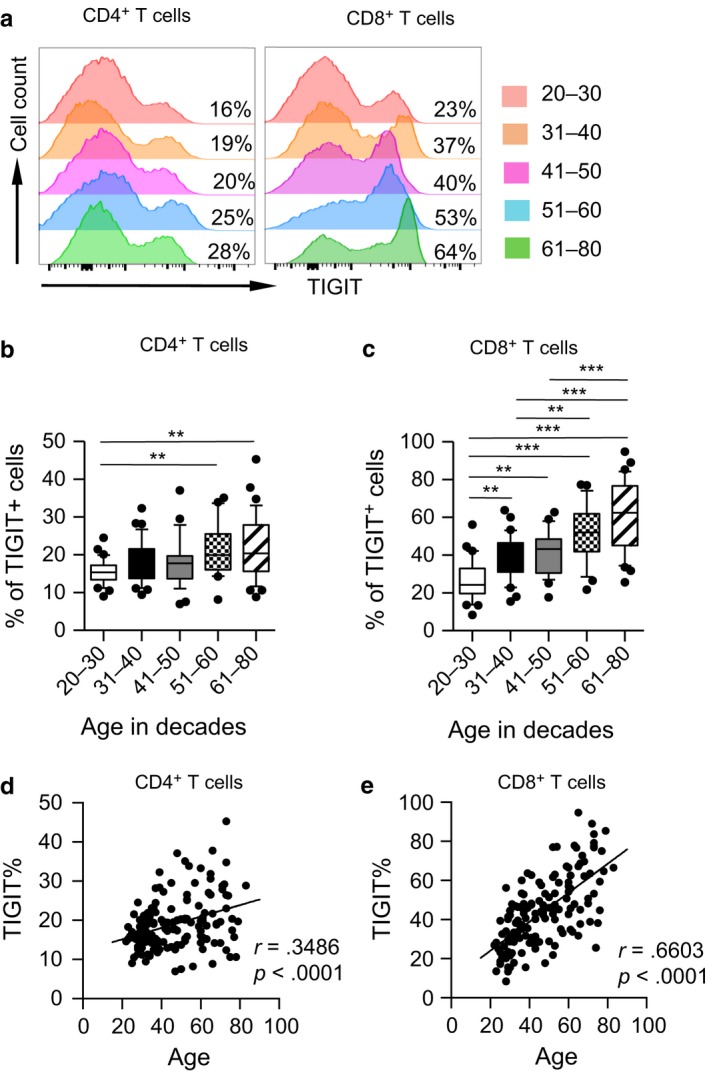
TIGIT is upregulated with age on CD8^+^ T cells from healthy individuals. Flow cytometry analysis of the surface expression of TIGIT was performed on PBMCs collected from healthy donors of different ages. (a) Representative histograms show the expression of TIGIT gated on CD4^+^ (left) and CD8^+^ T cells (right) from five healthy donors in different age groups. (b–c) Box plots of the percentages of TIGIT ^+^ cells on CD4^+^ and CD8^+^ T cells from healthy donors in different age groups (*n* = 27–37 each group). *p* Values were obtained by Kruskal–Wallis test followed by Dunn's multiple comparisons test [CD4^+^ T cells (left)] or one‐way ANOVA test followed by Tukey's multiple comparisons test [CD8^+^ T cells (right)]. (d–e) Correlation analysis of age and TIGIT expression on CD4^+^ T cells (d) and TIGIT ^+^ CD8^+^ T cells (e) from all healthy donors. Spearman's nonparametric test was used to test for correlations. **p < .01, ***p < .001

Strikingly, older adults had a higher frequency of TIGIT^+^ fractions among CD4^+^ and CD8^+^ T cells. Elevated frequencies of TIGIT^+^ fractions were more remarkable in CD8^+^ T cells than in CD4^+^ T cells (Figure 1a–c). Correlation analysis revealed that TIGIT^+^ cell frequencies among CD8^+^ T cells were significantly age correlated (*r* = .6603, *p* < .0001), whereas CD4^+^ T cells exhibited a weak correlation (*r* = .3486, *p* < .0001; Figure 1d and e).

### 2.2 TIGIT expression levels on different subsets of circulating T cells

**Figure 2:**
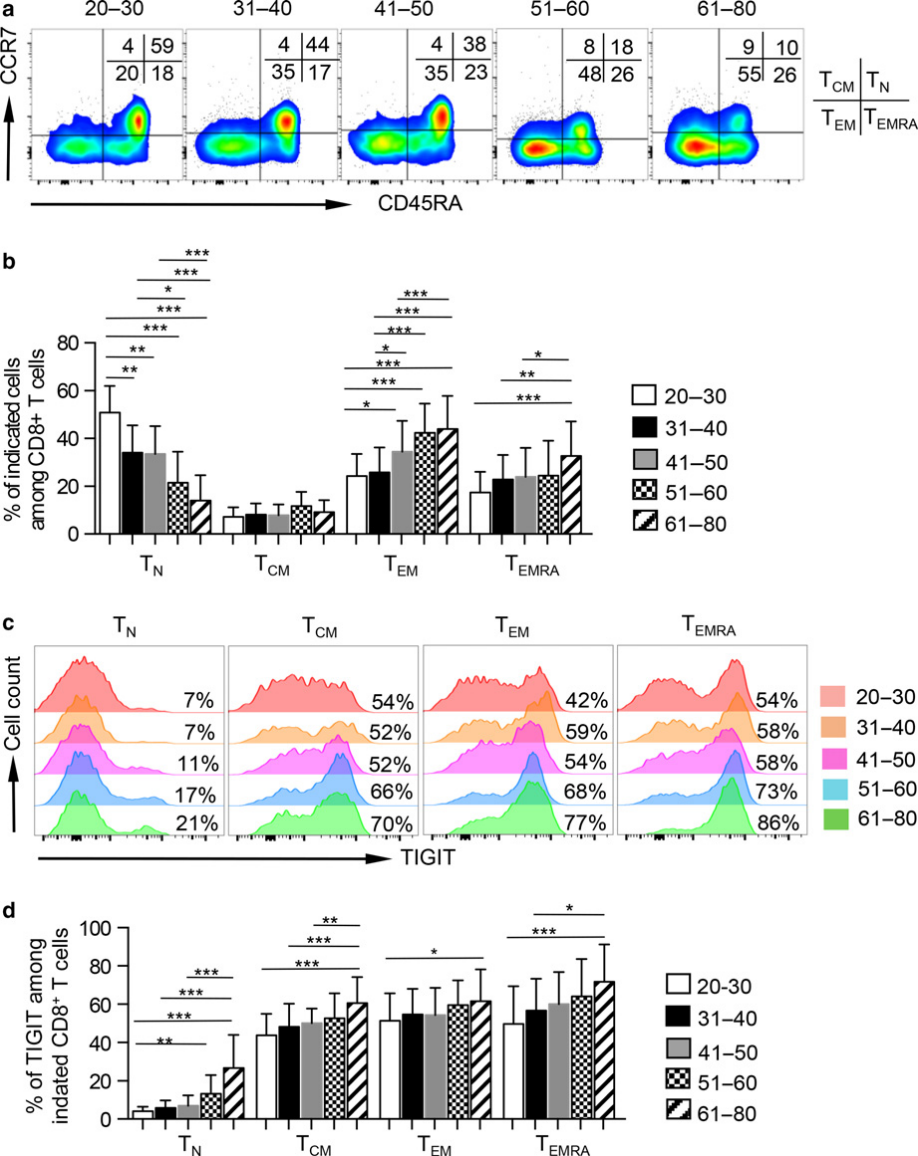
TIGIT is preferentially expressed on T_EM_ and T_EMRA_ cells, which are accumulated in the elderly. (a–b) Distribution of T_N_, T_CM_, T_EM_, and T_EMRA_ in CD8^+^ T cells from different age groups. Representative flow data gated on CD8 (a) and histograms (b) of the percentage of each subset in different age groups are shown (*n* = 27–37 each group). *p* Values were obtained by Kruskal–Wallis test followed by Dunn's multiple comparisons test (T_N_, T_CM_) or one‐way ANOVA test followed by Tukey's multiple comparisons test (T_EM_, T_EMRA_). (c–d) Expression of TIGIT on each subset (T_N_, T_CM_, T_EM_, and T_EMRA_) of CD8^+^ T cells. Representative flow data (c) and histograms (d) of the percentage of TIGIT expression on each subset of CD8^+^ T cells from five different age groups are shown. *p* Values were obtained by Kruskal–Wallis test followed by Dunn's multiple comparisons test (T_N_, T_EMRA_) or one‐way ANOVA test followed by Tukey's multiple comparisons test (T_CM_, T_EM_). *p < .05, **p < .01, ***p < .001

Previous studies showed higher numbers of antigen‐experienced T cells in the elderly (Jankovic, Messaoudi, & Nikolich‐Zugich, 2003); therefore, we investigated whether TIGIT was differently expressed on naïve T cells (T_N_, CCR7^+^CD45RA^+^) and more mature, antigen‐experienced subsets including central memory T cells (T_CM_, CCR7^+^CD45RA^−^), effector memory T cells (T_EM_, CCR7^−^CD45RA^−^), and terminally differentiated effector cells (T_EMRA_, CCR7^−^CD45RA^+^). In line with previous studies, the proportions of CD4^+^ and CD8^+^ T_N_ cells were dramatically decreased in the elderly compared with those in young and middle‐aged subjects, along with a significant increase in the proportions of CD4^+^ T_CM_ cells or CD8^+^ T_EM_ and T_EMRA_ cells (Figure 2a, b and S2a). The T_CM_, T_EM_, and T_EMRA_ subsets of CD8^+^ cells contained significantly higher frequencies of TIGIT^+^ cells than T_N_ cells independently of age (Figure 2c and d), indicating the upregulation of TIGIT in antigen‐experienced CD8^+^ cells. However, an elevated frequency of TIGIT^+^ fractions among CD8^+^ cells in the elderly was not only a result of the higher number of antigen‐experienced CD8^+^ cells. Although there was no difference of TIGIT expression on each T‐cell subset in CD4^+^ cells among young and older individuals (Figure S2b), each CD8^+^ T subset from older subjects expressed higher levels of TIGIT compared with those in their counterparts from young and middle‐aged subjects. A low percentage of T_N_ cells were TIGIT^+^ in young adults (approximately 2%), whereas >20% of T_N_ cells from subjects aged 60–80 years were TIGIT^+^. This indicated that elevated TIGIT^+^ frequency is a general character of T‐cell immunosenescence.

### 2.3 TIGIT^+^ CD8^+^ fractions from the elderly exhibited a phenotype of exhaustion and overactivation

**Figure 3:**
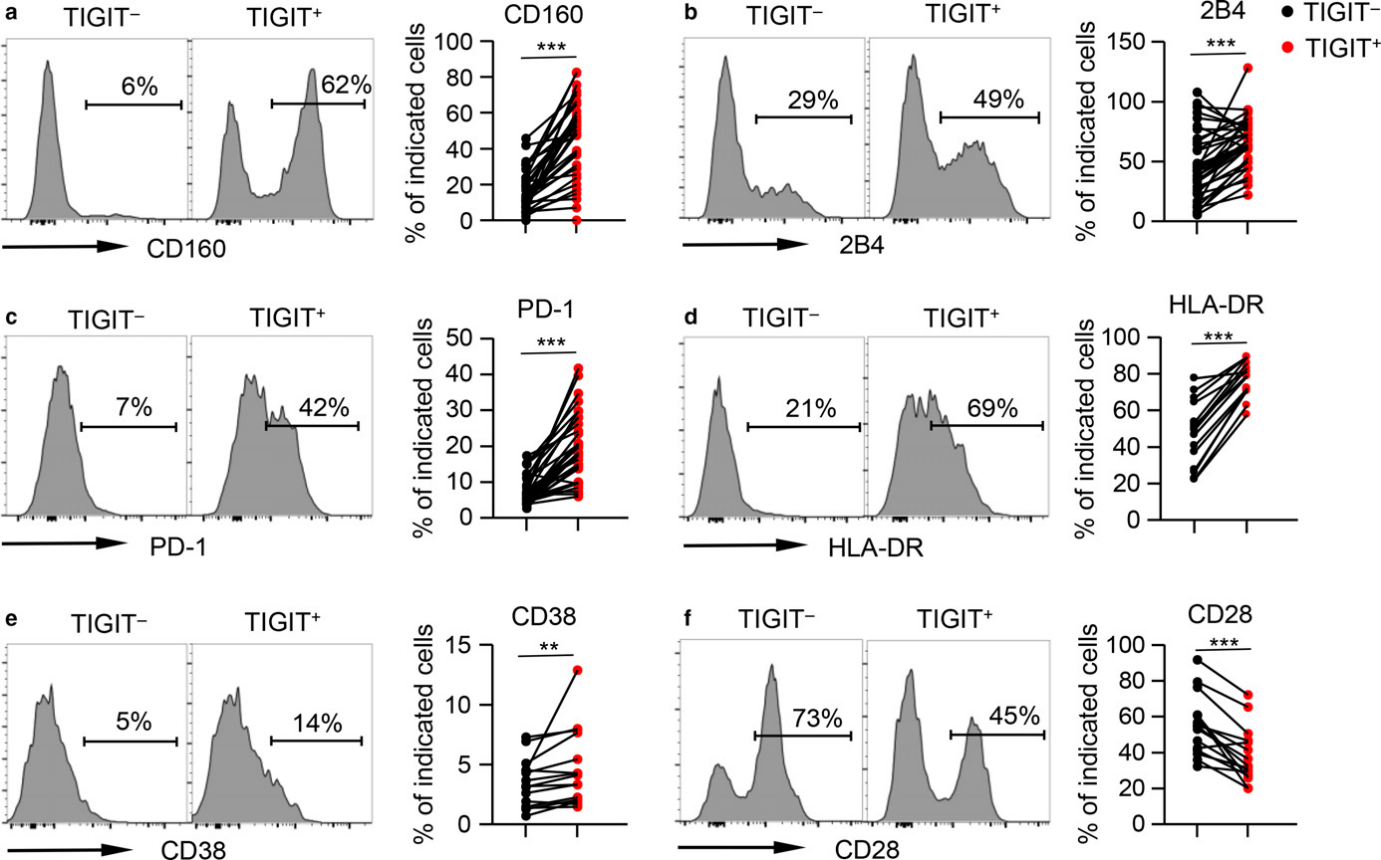
TIGIT expression is associated with the phenotypic profile of exhaustion and overactivation. Flow cytometry analysis of the expression of CD160 (a), 2B4 (b), PD‐1 (c), HLA‐DR (d), CD38 (e), and CD28 (f) on TIGIT ^−^ vs. TIGIT ^+^ CD8^+^ T cells from the elderly (61–80 years old, *n* = 37). Representative histograms (left) and plots (right) display the expression of the above receptors on TIGIT ^−^ vs. TIGIT ^+^ cells (gated with CD8^+^ T cells). *p* Values were obtained by paired *t* test or Wilcoxon matched‐pairs signed‐rank test. **p < .01, ***p < .001

To further determine whether TIGIT^+^CD8^+^ T cells in the elderly display a phenotype of exhaustion, we compared the expression levels of other inhibitory receptors on the TIGIT^+^ and TIGIT^−^ fractions of CD8^+^ T cells, including CD160, 2B4, PD‐1, BTLA, TIM‐3, and LAG‐3. The levels of CD160, 2B4, and PD‐1 were significantly higher on TIGIT^+^CD8^+^ T cells than on TIGIT^−^CD8^+^ T cells in the elderly (Figure 3a–c). Similar results were obtained on TIGIT^+^CD8^+^ T cells from the young and middle‐aged groups (Figure S3a–c). Analysis of the entire cohort revealed a close correlation between TIGIT expression and CD160 or 2B4 levels (Figure S3d–f). By contrast, the expression levels of TIM‐3, LAG‐3, and BTLA were comparable between TIGIT^+^CD8^+^ T cells and TIGIT^−^CD8^+^ T cells in young and old individuals (Figure S3g–i), and no significant correlation was observed between the expression of TIGIT and BTLA, TIM‐3, or LAG‐3 (Figure S3j–l).

As T‐cell exhaustion with a stepwise loss of function is a consequence of overactivation of T cells caused by high antigenic stimulation, we next assessed the activation status of TIGIT^+^CD8^+^ T cells by measuring the expression of CD38^+^ and HLA‐DR^+^. The results showed that the frequency of HLA‐DR^+^ cells was significantly higher in the TIGIT^+^CD8^+^ fraction than in TIGIT^−^CD8^+^ T cells regardless of age (Figure 3d and S4a). In addition, TIGIT^+^CD8^+^ T cells from the elderly displayed higher frequencies of CD38^+^ than TIGIT^−^ CD8^+^ T cells (Figure 3e and S4b). Along with an increase in activated cells, TIGIT^+^CD8^+^ T cells displayed lower levels of CD28 than TIGIT^−^CD8^+^ T cells, suggesting a reduced antigen‐mediated T‐cell response (Figure 3f and S4c). Collectively, these data suggested that aged TIGIT^+^CD8^+^ T cells remained in an overactivated and consequently exhausted status.

### 2.4 TIGIT^+^CD8^+^ T cells comprised a high number of terminal exhausted T‐bet^dim^Eomes^hi^ cells

**Figure 4:**
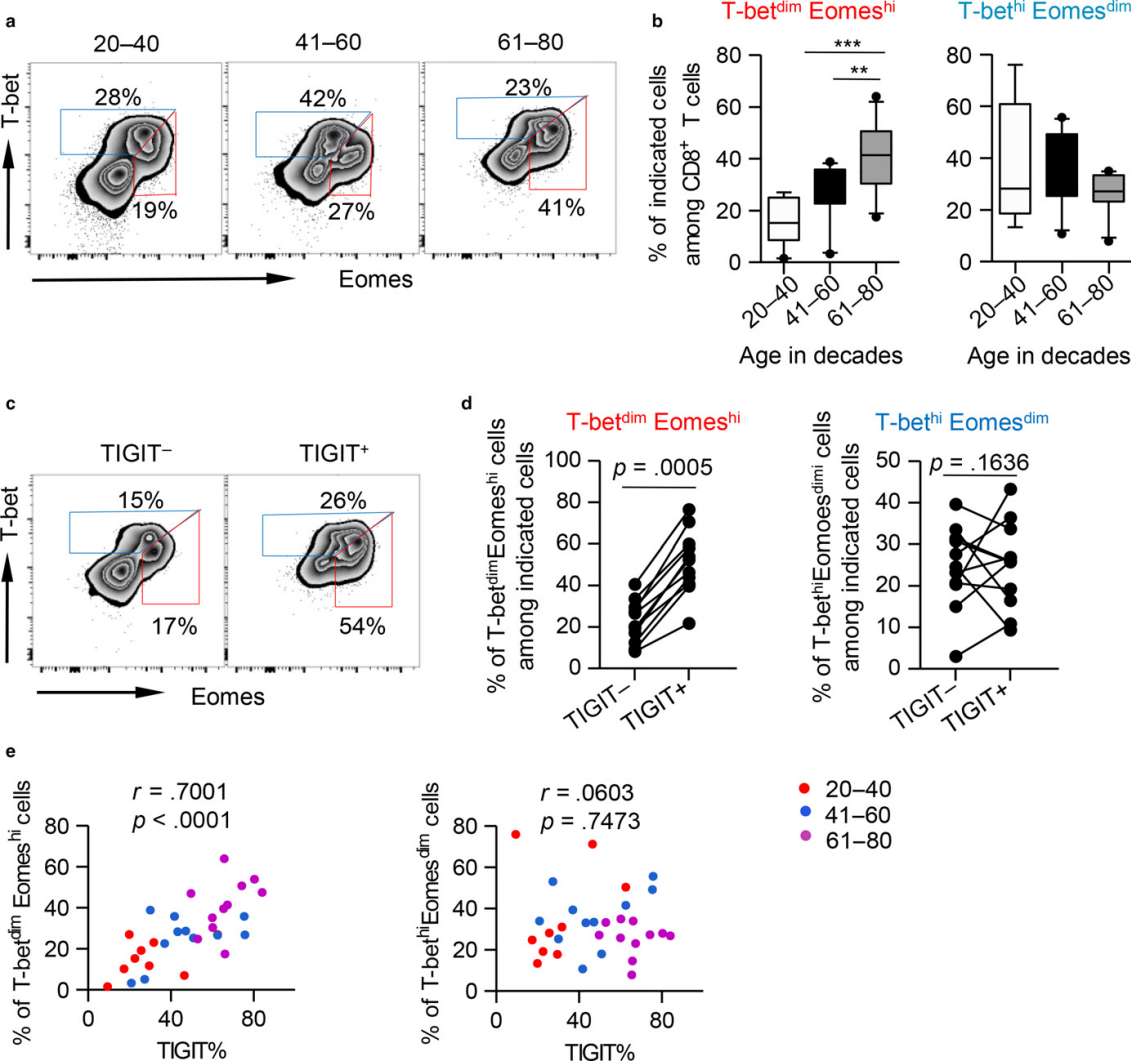
Aged TIGIT ^+^ CD8^+^ T cells exhibited an elevated T‐bet^dim^Eomes^hi^ population and a decreased T‐bet^hi^Eomes^dim^ population. (a–b) Representative flow data (a) and box plots (b) of the percentage of T‐bet^dim^Eomes^hi^ and T‐bet^hi^Eomes^dim^ cells among CD8^+^ T cells from different age groups (*n* =) are shown. *p* Values were obtained by Kruskal–Wallis test followed by Dunn's multiple comparisons test or one‐way ANOVA test followed by Tukey's multiple comparisons test. (c) Gating strategy to distinguish the T‐bet^dim^Eomes^hi^ (red) from the T‐bet^hi^Eomes^dim^ (blue) population in TIGIT ^−^ and TIGIT ^+^ CD8^+^ T cells in the elderly (61–80 years old, *n* = 11). (d) Plots of the percentage of T‐bet^dim^Eomes^hi^ (left) and T‐bet^hi^Eomes^dim^ (right) cells among TIGIT ^−^ and TIGIT ^+^ CD8^+^ T cells in the elderly. Statistical analysis was performed using paired t test. (e) Correlation analysis of TIGIT expression and the frequencies of T‐bet^dim^Eomes^hi^ and T‐bet^hi^Eomes^dim^ cells among CD8^+^ T cells of all ages. Pearson's test was used to test for correlations. **p < .01, ***p < .001

To further characterize the intrinsic regulation of TIGIT^+^CD8^+^ T cells, we examined the expression of T‐bet and eomesodermin (Eomes), two key transcription factors governing CD8^+^ T‐cell exhaustion. Previous reports indicated that T‐bet^dim^Eomes^hi^ CD8^+^ T cells represent a terminal exhausted phenotype, whereas T‐bet^hi^Eomes^dim^ cells retain some residual T‐cell functions (Paley et al., 2012). We observed that the percentages of T‐bet^dim^Eomes^hi^ CD8^+^ T cells from the elderly were significantly higher than those from middle‐aged and young adults (Figure 4a and b). In addition, TIGIT^+^CD8^+^ T cells from the elderly contained higher percentages of T‐bet^dim^Eomes^hi^ cells than TIGIT^−^CD8^+^ T cells (Figure 4c and d). Accordingly, TIGIT expression on CD8^+^ T cells from all ages was positively correlated with T‐bet^dim^Eomes^hi^ frequencies but not with T‐bet^hi^Eomes^dim^ cell frequencies (Figure 4e). Therefore, TIGIT^+^CD8^+^ T cells comprised a high number of terminally differentiated cells.

### 2.5 TIGIT^+^CD8^+^ T cells from the elderly showed functional defects

**Figure 5:**
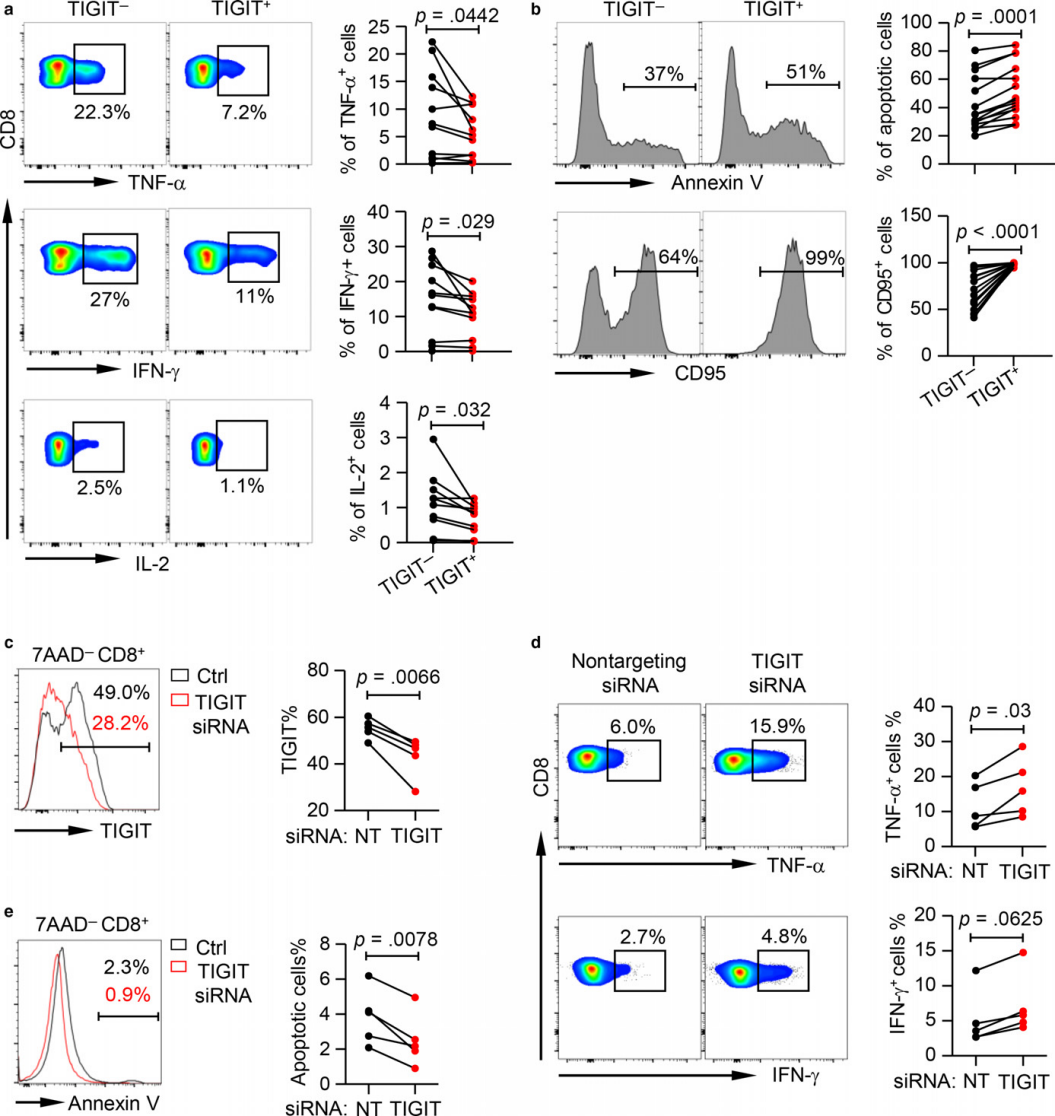
Aged TIGIT ^+^ CD8^+^ T cells exhibit high susceptibility to apoptosis and defects in cytokine production, which can be reversed by TIGIT knockdown. (a) Intracellular staining for TNF‐α, IFN‐γ, and IL‐2 in TIGIT ^−^ and TIGIT ^+^ CD8^+^ T cells from the elderly (61–80 years old, *n* = 11) upon in vitro anti‐CD3/anti‐CD28 stimulation. Shown are representative flow data (left) and summary data (right) for TNF‐α, IFN‐γ, and IL‐2, respectively. *p* Values were obtained by paired t test. (b) Percentage of apoptotic cells (7AAD ^−^Annexin V^+^) and expression of CD95 in TIGIT ^−^ and TIGIT ^+^ CD8^+^ T cells from the elderly (61–80 years old, *n* = 11). Representative histograms (left) and plots of the percentage of apoptotic cells (right) are shown. *p* values were obtained by paired *t* test (CD95) or Wilcoxon matched‐pairs signed‐rank test (Annexin V). (c–e) Purified CD8^+^ T cells from the elderly (*n* = 5) were transfected with indicated SMARTpool siRNA. After cultured in vitro for 3 days, intracellular cytokine production in response to anti‐CD3 stimulation and anti‐CD28 stimulation and the susceptibility of apoptosis were evaluated by flow cytometry. (c) TIGIT knockdown significantly downregulated TIGIT expression on CD8^+^ T cells. Representative flow data (left) and a plot of TIGIT expression (right) on CD8^+^ T cells transfected with nontargeting siRNA vs. TIGIT siRNA are shown. (d) TIGIT knockdown increases cytokine production. Representative flow data (left) and plots (right) of the percentage of TNF‐α^+^ and IFN‐γ^+^ cells are shown. (e) TIGIT knockdown improves apoptosis. Representative histogram (left) and plot (right) of Annexin V expression in 7AAD ^−^ CD8^+^ T cells are shown. *p* values were obtained by paired *t* test (TIGIT, TNF‐α, IFN‐γ) or Wilcoxon matched‐pairs signed‐rank test (Annexin V)

To further characterize the function of TIGIT^+^CD8^+^ T cells from aging individuals, we assessed cytokine release upon in vitro stimulation with anti‐CD3 and anti‐CD28 antibodies. Strikingly, TIGIT^+^CD8^+^ T cells and TIGIT^−^CD8^+^ T cells from young and middle‐aged groups produced comparable levels of TNF‐α, IFN‐γ, and IL‐2, suggesting that high TIGIT levels did not affect T‐cell dysfunction in young and middle‐aged individuals (Figure S5a–c). However, TIGIT^+^CD8^+^ T cells from the elderly produced significantly lower amounts of TNF‐α, IFN‐γ, and IL‐2 than TIGIT^−^CD8^+^ T cells (Figure 5a). Moreover, TIGIT^+^CD8^+^ T cells exhibited a high susceptibility to apoptosis, as indicated by increased frequencies of 7AAD^−^Annexin V^+^ and CD95^+^ fractions in subjects of all ages (Figure 5b and S5d,e). To study a direct effect of TIGIT in T‐cell dysfunction, we performed a TIGIT knockdown experiment using SMARTpool siRNA and individual siRNAs and evaluated the T‐cell functions upon TIGIT knockdown (Figure 5c and S6a). We found a significant increased cytokine release and less apoptosis in CD8^+^ T cells from elderly subjects upon TIGIT knockdown (Figure 5d, e and S6b, c). This important data demonstrate the suppressive effect of TIGIT in T‐cell function in the elderly.

We further assayed the proliferation ability of TIGIT^+^CD8^+^ T cells from the elderly by measuring ki‐67 expression. TIGIT^+^CD8^+^ T cells exhibited significantly higher levels of ki‐67 expression than TIGIT^−^CD8^+^ T cells (Figure S7a). Moreover, TIGIT^+^CD8^+^ T cells from the elderly displayed higher levels of CD107a, Granzyme B, and perforin (Figure S7b–d), indicating that these cells have a greater nonspecific killing potential. Similar results were obtained in TIGIT^+^CD8^+^ T cells from the young and middle‐aged groups (data not shown). These data demonstrate that aged TIGIT^+^CD8^+^ T cells possess impaired function to some degree by displaying a low capacity for cytokine production and a high susceptibility to apoptosis while retaining their proliferation capacity and killing potential.

### 2.6 CD226 was downregulated on aged TIGIT^+^CD8^+^ T cells

**Figure 6:**
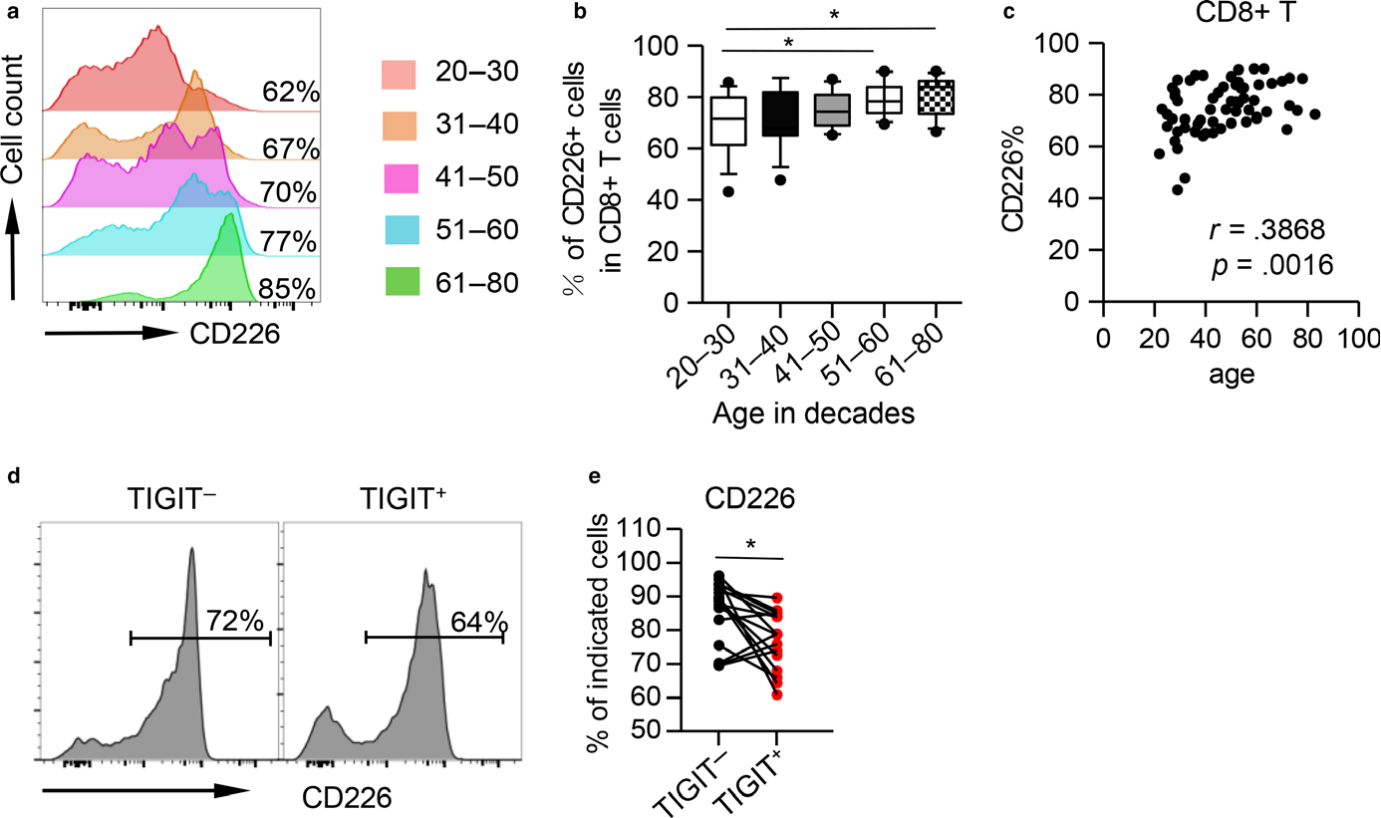
CD226 expression on aged CD8^+^ T cells is inversely associated with the expression of TIGIT. (a–b) Flow cytometry analysis of PBMCs from different age groups for CD226 expression on CD8^+^ T cells. Representative flow data and a box plot of the percentage of CD226^+^ cells among CD8^+^ T cells from different age groups are shown (*n* = 11–16 each group). *p* values were obtained using the Kruskal–Wallis test followed by Dunn's multiple comparisons test. (c) Correlation analysis between age and CD226 expression on CD8^+^ T cells. Spearman's nonparametric test was used to test for correlations. (D–E) Representative histogram data (d) and plot data (e) of CD226 expression on TIGIT ^−^ vs. TIGIT ^+^ CD8^+^ T cells from the elderly (61–80 years old, *n* = 11) are shown. *p* values were obtained using the Wilcoxon matched‐pairs signed‐rank test. *p < .05

Recent studies suggest that TIGIT exerts inhibitory effects by competing with its costimulatory counterpart, CD226, for their common ligand, CD155. To determine whether CD226 is involved in TIGIT‐mediated immune suppression during aging, we evaluated the expression of CD226 on CD8^+^ T cells. Consistent with the upregulation of TIGIT, CD226 levels increased gradually with age (Figure 6a and b). In addition, the expression levels of CD226 were positively correlated with age (*r* = .3868, *p* = .0016, Figure 6c). However, the expression of CD226 was significantly lower on aged TIGIT^+^CD8^+^ T cells than on TIGIT^−^CD8^+^ T cells (*p* < .0001, Figure 6d and e), whereas CD226 expression on young T cells was comparable between TIGIT^+^ and TIGIT^−^CD8^+^ T cells. This could provide an explanation for the differences in cytokine production between the young, middle‐aged, and elderly groups (Figure S8).

## 3 DISCUSSION

It has been demonstrated that upregulation of co‐inhibitory receptors is an important feature for T‐cell exhaustion in chronic infection and tumors (Pauken & Wherry, 2015). Most recently, elevation of PD‐1 and TIM‐3 was observed in aging mice (Lee et al., 2016). As senescent and exhausted T cells exhibit similar features of immune dysfunction, it has been speculated that these two processes share common mechanisms. Studies show that a number of co‐inhibitory receptors, including PD‐1, TIM‐3, LAG‐3, and CTLA‐4, are associated with impaired T‐cell function in aged mice (Channappanavar et al., 2009; Decman et al., 2012; Shimada et al., 2009). However, we did not observe a correlation between these co‐inhibitory receptors and aging in humans. Instead, we found a significant upregulation of TIGIT expression on T cells from elderly healthy donors compared with that from young individuals. The difference was more prominent in CD8^+^ T cells. TIGIT^+^ CD8^+^ T cells expressed high levels of other inhibitory receptors and displayed multiple functional defects, including reduced cytokine production and susceptibility to apoptosis. These data suggest that TIGIT is a biomarker and elucidate a potential mechanism of T‐cell senescence. To the best of our knowledge, this is the first evidence linking TIGIT to immunosenescence.

Both senescent and exhausted T cells are dysfunctional. However, they differ in their immune phenotypes: severely exhausted CD8^+^ T cells exhibit lower expression of KLRG1 and CD57, which are markers that are elevated in senescence (Angelosanto, Blackburn, Crawford, & Wherry, 2012). Previous studies show that the upregulation of PD‐1 and TIGIT on antigen‐specific CD8^+^ T cells contribute to their exhaustion in chronic viral infection and cancer (Chauvin et al., 2015; Johnston et al., 2014; Kong et al., 2016). In the present study, we demonstrated a tight correlation between TIGIT and aging. Thus, TIGIT might be a unique marker of both T‐cell exhaustion and senescence. It is worth noting that findings in animal models do not necessarily represent the phenomenon in humans (den Braber et al., 2012). Several inhibitory receptors such as PD‐1, TIM‐3, LAG‐3, and CTLA‐4 are upregulated in aged mice. However, this pattern was not observed in human samples from elderly individuals in the present study. The discrepancy highlights the limitations associated with the use of animal models in studies of immune senescence.

Another interesting issue in the present study was that the upregulation of TIGIT in the cohort started early and became worse with age, which indicated that T‐cell senescence exists not only in the elderly but also in young individuals. Although senescence is thought to be associated with the physiological aging process, chronic activation, stimulation, or damage may accelerate T‐cell senescence. A high percentage of senescent T cells is observed in young patients with chronic viral infections and autoimmune diseases (Pawelec & Solana, 2001; Vallejo, Weyand, & Goronzy, 2004). It remains unclear whether the age‐dependent upregulation of TIGIT is associated with a physiological process or pathogenic stimulation. It has been reported that pathogenic stimulation‐induced TIGIT upregulation is frequent with increases of other co‐inhibitory receptors, especially PD‐1 (Chauvin et al., 2015; Johnston et al., 2014). However, this was not the case in the present study. Therefore, it is possible that TIGIT‐associated T‐cell senescence is a consequence of physiological stimulation. Moreover, despite TIGIT expression in the young and middle‐aged, TIGIT^+^CD8^+^ T cells in the elderly were more dysfunctional than the population from the young and middle‐aged groups, especially regarding defective cytokine production. The difference in cytokine production between the groups was associated with the expression pattern of CD226, the costimulatory counterpart of TIGIT.

Recent studies demonstrated that TIGIT suppresses antiviral and antitumor CD8 T‐cell immunity. Our novel observation that TIGIT is highly expressed on senescent T cells led us to speculate that TIGIT contributes to the functional defect of these T cells and subsequently increases the susceptibility to infection or cancer. In fact, elderly patients are susceptible to a large number of infectious diseases. The incidence of community‐acquired pneumonia and urinary tract infection is threefold and 20‐fold higher, respectively, in elderly patients than in young individuals (Gavazzi & Krause, 2002). The risk of listeria meningitis, a rare disease, is also significantly increased in elderly patients (Choi, 2001). Similarly, TIGIT upregulation in the elderly may lead to age‐related T‐cell dysfunction and the loss of immune surveillance for cancer.

In fact, a thorough understanding of the antigen specificity would be helpful to clarify age‐related T‐cell dysfunction. However, in contrast to well‐defined antigen‐specific systems in mouse models, the study of antigen‐specific T‐cell function in human has been challenging due to high clinical diversities (different environmental exposure), limited number of defined specific antigens, and the low number of T cells reactive to each specific epitope. In addition, previous studies of immunosenescence have demonstrated that accumulation of aged CD8^+^ T cells is a result of the exposure to a broad range of antigens over the lifespan (Bunztman, Vincent, Krovi, Steele, & Frelinger, 2012; Lee et al., 2016). Thus, our capability of investigating CD8^+^ T cells specific to immunosenescence‐related antigens is extremely limited. Our functional studies were mostly performed using CD8^+^ T cells from elderly donors, which are potentially highly enriched with T cells responded to immunosenescence‐related antigens. Therefore, the results of functional assay in our experiments are more representative for the effect of TIGIT in immunosenescence‐related antigen‐specific T cells.

Two transcription factors, T‐bet and EOMES, were reported to cooperate to regulate exhausted CD8^+^ T cells in a mouse model of chronic viral infection (Paley et al., 2012). Based on the expression level of these two transcription factors, exhausted CD8^+^ T cells are divided into two subsets: T‐bet^hi^Eomes^dim^ cells, which retain some proliferative capacity, and Eomes^hi^T‐bet^dim^ terminal progeny, which express higher levels of inhibitory receptors and exhibit low proliferative capacity. Persistent antigen stimulation causes T‐bet^hi^Eomes^dim^ to lose T‐bet expression, undergo proliferation, and convert to Eomes^hi^T‐bet^dim^ subset (Buggert et al., 2014; Moro‐Garcia et al., 2012; Odorizzi, Pauken, Paley, Sharpe, & Wherry, 2015). In the present study, we observed an increase in the Eomes^hi^T‐bet^dim^ terminal population, whereas T‐bet^hi^Eomes^dim^ progenitor cells decreased in aged CD8^+^ T cells. In addition, the percentage of Eomes^hi^T‐bet^dim^ cells was significantly higher in TIGIT^+^ CD8^+^ T cells than in TIGIT^−^ cells. Interestingly aged TIGIT^+^ CD8^+^ T cells showed decreased cytokine production but potent killing ability, a unique feature of Eomes^hi^T‐bet^dim^ terminal progeny. Thus, aged TIGIT^+^CD8^+^ T cells are a mixed population with a predominance of Eomes^hi^T‐bet^dim^ terminal cells with a hierarchical loss of function.

Unlike exhausted T cells, TIGIT^+^ CD8^+^ T cells in the elderly expressed high levels of intracellular Granzyme B and perforin and displayed higher cytotoxic capacity. Contrary to the traditional concept, recent studies demonstrated that senescent cells are not dormant, but rather metabolically active, and they express a vast number of secreted proteins, which is in line with our findings. This phenotype, which is referred to as the “senescence‐associated secretory phenotype,” provides a strong link between senescence and inflammation (Lasry & Ben‐Neriah, 2015; Rodier et al., 2009). Of note, aging is also associated with an enhanced inflammatory response, another feature of immunosenescence (Raj et al., 2015; Shaw et al., 2013; Williams, Jose, Brown, & Chambers, 2015; Yung & Julius, 2008). Thus, senescence‐related inflammation is thought to underlie the increased incidence of autoimmune diseases in the elderly. A thorough understanding of how the stress response to aging impacts the immune system of the elderly would be helpful to determine their increased disease spectrum and disease susceptibility.

An interesting finding in the present study is that TIGIT^+^ CD8^+^ T cells from the elderly exhibited significantly higher levels of proliferation than TIGIT^−^ CD8^+^ T cells. This is somewhat surprising as cellular senescence is generally considered as a stage with irreversible arrest of cell proliferation (Campisi & d'Adda di Fagagna, 2007). However, it is worth to note that unlike senescence in other cell types, T‐cell immunosenescence has unique characteristics. Memory T cells, which is predominant in the elderly, can be long‐term lived and undergo self‐renewal, an important feature for stem cells (Fearon, Manders, & Wagner, 2001; Gattinoni, Klebanoff, & Restifo, 2012; Luckey et al., 2006). It has been known that the ability of stem cells to self‐renew and long‐term live is tightly regulated by cell cycle process (Orford & Scadden, 2008). For example, maintenance of hematopoietic stem cells in G0 phase is crucial to ensure a potent, self‐renewable system that supports lifelong multilineage hematopoiesis (Zeng, Yucel, Kosan, Klein‐Hitpass, & Moroy, 2004). In contrast to memory phase, T cells undergoing immunosenescence are not able to maintain into G0 phase in the cell cycle thus subsequently lose the capacity of self‐renewal and long‐term living. Therefore although TIGIT^+^ CD8 T cells are highly proliferative likely due the early stage of T‐cell activation, their immunosenescence fate determined by the TIGIT expression will lead them leave G0 and enter the next cell cycle stage, eventually lose the ability to self‐renew.

A key unresolved issue is the mechanism mediating the upregulation of TIGIT on aged T cells. In chronic viral infections and cancer, the co‐inhibitory receptors PD‐1, TIM‐3, and LAG‐3 are induced upon persistent antigen stimulation. In the present study, naïve T cells were reduced and memory T cells were increased in the elderly, indicating the contribution of lifelong encounters with pathogens and persistent antigenic stimulation. Previous studies including ours showed that the majority of TIGIT^+^CD8^+^ T cells were T_CM_, T_EM_, and T_EMRA_, indicating that they are antigen‐experienced cells (Chew et al., 2016; Kong et al., 2016). This was further supported by the excessive activation of T cells with high expression of CD38 and HLA‐DR. Consistently, in vitro stimulation with anti‐CD3/28 was reported to upregulate TIGIT expression on T cells. Furthermore, aging is associated with an increased inflammatory response, another feature of immunosenescence. Several studies suggest that abnormalities in the aging immune system can be attributed not only to T‐cell dysfunction but also to the concomitant increase in proinflammatory cytokines, including IL‐6, IL‐23, TNF‐α, sIL‐2 receptor, IL‐8, MCP‐1, MIP‐1a, and RANTES (El Mezayen, El Gazzar, Myer, & High, 2009; Yung & Julius, 2008). In fact, several common gamma‐chain (γ‐chain) cytokines such as IL‐2, IL‐15, and IL‐21 have been shown to directly upregulate TIGIT expression on CD8^+^ T cells from HIV‐infected individuals (Chew et al., 2016). We also observed that another γ‐chain cytokine, IL‐7, induced a significant upregulation of TIGIT on T cells from healthy individuals as well as IL‐2 (data not shown). Taken together, these findings indicate that during aging, persistent self‐ or environmental antigens and inflammatory cytokines might collectively contribute to the age‐related upregulation of TIGIT and concomitant T‐cell dysfunction.

In conclusion, the present study demonstrated that TIGIT is a prominent negative immune regulator involved in immunosenescence. This novel finding is highly significant, as targeting TIGIT might be an effective strategy to improve the immune response and decrease age‐related comorbidities.

## 4 EXPERIMENTAL PROCEDURES

### 4.1 Subjects

The study was approved by the Committee of Ethics at Beijing Ditan Hospital, Capital Medical University, Beijing, China. The study subjects were healthy volunteers aged 18–80 years (84 men and 80 women) recruited between February 2016 and July 2016. Gender was evenly distributed in each group. Subjects who tested positive to human immunodeficiency virus (HIV) infection, hepatitis viral infections, systemic infection, connective tissue disease, cancer, or abnormal tumor markers including alpha fetoprotein (AFP), carcinoembryonic antigen (CEA), carbohydrate antigen (CA‐199), CA‐153, and CA‐125 were excluded.

### 4.2 Isolation of peripheral blood mononuclear cells (PBMCs)

Peripheral blood samples were collected from healthy volunteers. Blood was diluted 1:1 with phosphate‐buffered saline before separation of PBMCs by Ficoll‐Paque (Amersham Pharmacia Biotech, Sweden) density gradient centrifugation. Cells were cryopreserved in fetal bovine serum (GIBCO, Grand Island, NY, USA), supplemented with 10% DMSO, and stored in liquid nitrogen.

### 4.3 Immunofluorescence staining and flow cytometric analysis

Peripheral blood mononuclear cells were incubated with directly conjugated antibodies for 30 min at 4°C. The cells were then washed before flow cytometric analysis. Antibodies used were anti‐human CD3‐BV786, CD4‐APC‐H7, CD8‐BV510 or CD8‐BV421, CD45RA‐AF700, CCR7‐BV421, CD95‐PE‐CF594, PD‐1‐BV711, BTLA‐BV650, TIM‐3‐BV650, CD160‐AF488, 2B4‐FITC, CD107a BV421 (BD Biosciences, San Diego, CA, USA), CD226‐FITC, Granzyme B‐AF700, perforin‐PE (BioLegend, San Diego, CA, USA), TIGIT‐PE‐Cy7, and LAG‐3‐APC (Ebioscience, San Diego, CA, USA) and the corresponding isotype controls. Data acquisition was performed on an LSR Fortessa flow cytometer (BD Biosciences), and data analysis was performed using FlowJo Software (Tree Star, Ashland, OR, USA).

### 4.4 In vitro stimulation and intracellular staining

Peripheral blood mononuclear cells were cultured in RPMI‐1640 medium (GIBCO, Grand Island, NY, USA) containing 10% FBS and stimulated with anti‐CD3/CD28 (2 μg/mL and 5 μg/mL; Ebioscience) or phorbol myristate acetate (PMA)/ionomycin (50 ng/mL and 1 μg/mL), plus Golgiplug (BD Biosciences) for 5 h. The cells were then surface‐stained with CD3‐BV786, CD4‐APC‐H7, CD8‐BV421, PD‐1‐BV711, or TIGIT‐PE‐Cy7, and intracellularly stained with IFN‐γ‐AF700, TNF‐α‐FITC, or IL‐2‐PE (BD Biosciences) antibodies. For ki67, perforin, T‐bet, or Eomes staining, PBMCs were surface‐stained with CD3‐BV786, CD4‐ APC‐H7, CD8‐BV650, PD‐1‐BV711, or TIGIT‐PE‐Cy7, and intracellularly stained with perforin‐APC, ki67‐FITC, Granzyme B‐AF700, T‐bet BV421, or Eomes PE (BD Biosciences) antibodies. A Fixable Viability Dye eFluor^®^ 506 (Ebioscience) was used to assess cell viability.

### 4.5 Apoptosis analysis

Apoptosis rates were measured using a FITC Annexin V Apoptosis Detection Kit (BioLegend) following the manufacturer's instructions, in combination with markers for T cells. Samples were analyzed by flow cytometry.

### 4.6 siRNA transfection

As TIGIT was reported to be able to suppress T cells directly by delivering intrinsic inhibition in the absence of APCs/ligands (Joller et al., 2011), purified CD8^+^ T cells were used for transfection experiments here as described before (Kong et al., 2016; Lozano, Dominguez‐Villar, Kuchroo, & Hafler, 2012). Briefly, CD8^+^ T cells were isolated from PBMCs using positive selection with human CD8 MicroBeads (Miltenyl Biotec, Teterow, Germany). We transfected 1 μm SMARTpool Accell TIGIT siRNA or Accell nontargeting pool siRNA, as well as 2 μm indicated single TIGIT siRNA or single nontargeting siRNA in a 96‐well tissue culture plate with Accell siRNA delivery media for 72 h (GE Dharmacon, Lafayette, CO, USA). TIGIT expression, Annexin V staining, and cytokine production were measured by flow cytometry. SMARTpool Accell TIGIT siRNA includes a mixture of four individual siRNA species targeting distinct sequences of TIGIT gene. Accell nontargeting pool contains four siRNAs designed to have minimal targeting of known genes in human, mouse, and rat cells. Sequence information of the siRNA pools and individual siRNA used from Dharmacon are described in Table S1.

### 4.7 Statistical analysis

Data are expressed as the mean ± standard deviation (*SD*). GraphPad5 (GraphPad Software, La Jolla, CA, USA) or SPSS (IBM Corporation, New York, NY, USA) were used for statistical calculations. The normality of each variable was evaluated using the Kolmogorov–Smirnov test. In cases of two normally distributed data, the comparison of variables was performed using unpaired, or paired where specified, two‐tailed Student's *t* tests for unpaired and paired data, respectively. One‐way ANOVA test followed by Tukey's multiple comparisons test was performed for comparing two more independent samples. When the data were not normally distributed, the comparison of variables was performed with a Mann–Whitney U test or a Wilcoxon matched‐pairs signed‐rank test for unpaired and paired data, respectively. For comparing two more independent samples, a Kruskal–Wallis test followed by Dunn's multiple comparisons test was applied. Comparisons of patient characteristics were analyzed using Fisher's exact test (categorical variables) or Kruskal–Wallis test (continuous variables). Pearson's or Spearman's correlation coefficients were used to evaluate correlations for normally or non‐normally distributed data, respectively. For all analyses, *p* values <.05 were considered statistically significant.

## CONFLICT OF INTEREST

The authors declare no conflict of interests.

## AUTHOR CONTRIBUTIONS

S.Y. and B.W. performed the experiments and analyzed the data. R.S., Y.H., D.W., Y.L., Y.J., L.X., and Y.M. collected samples and performed the experiments. H.Zh. participated in the critical review of the manuscript. Y.K. designed the experiments, analyzed the data, and wrote the manuscript. H.Z. designed the experiments and wrote the manuscript.
