# Supplementary material for: T‐cell Immunoglobulin and ITIM Domain Contributes to CD8+ T‐cell Immunosenescence
Source: Aging Cell. 2018 Jan 19;17(2):e12716. doi: 10.1111/acel.12716 (PMC5847879; doi:10.1111/acel.12716)
Supplement: Supplementary file 1 [file ACEL-17-e12716-s001.pdf]

Supporting Information

Fig. S1 Expression of inhibitory receptors on $CD8^+$ T cells of healthy individuals from different age groups

Flow cytometry analysis of the surface expression of PD-1 (A), CTLA-4 (B), TIM-3 (C), and LAG-3 (D) on PBMCs collected from healthy donors in different age groups (n = 27–37 each group). P values were obtained using the Kruskal-Wallis test followed by Dunn's multiple comparisons test or one-way ANOVA test followed by Tukey's multiple comparisons test. *P < 0.05, **P < 0.01, ***P < 0.001

Fig. S2 Distribution of $CD4^+$ T cell subsets and TGIT expression on each subset from different age groups

(A) Distribution of T_N, T_{CM}, T_{EM}, and T_{EMRA} in $CD4^+$ T cells from different age groups (n = 27–37 each group). P values were obtained by one-way ANOVA test followed by Tukey's multiple comparisons test (T_N, T_{CM}) or Kruskal–Wallis test followed by Dunn's multiple comparisons test (T_{EM}, T_{EMRA}). (B) Expression of TIGIT on each subset (T_N, T_{CM}, T_{EM}, and T_{EMRA}) of $CD4^+$ T cells. P values were obtained by Kruskal-Wallis test followed by Dunn's multiple comparisons test (T_N, T_{EMRA}) or one-way ANOVA test followed by Tukey's multiple comparisons test (T_{CM}, T_{EM}). *P < 0.05, **P < 0.01, ***P < 0.001.

Fig. S3 TIGIT expression is associated with certain inhibitory receptors

Flow cytometry analysis of the expression of CD160 (A), 2B4 (B), PD-1 (C), TIM-3 (G), LAG-3 (H), and BTLA (I) on $TIGIT^-$ vs. $TIGIT^+$ $CD8^+$ T cells from different age

groups (n = 27–37 each group). P values were obtained using the paired t test or Wilcoxon matched-pairs signed rank test. Correlation analysis of TIGIT and expression of CD160 (D), 2B4 (B), PD-1 (E),TMI-3 (J), LAG-3 (K), and BTLA (L). Pearson’s or Spearman’s non-parametric test was used to test for correlations. **P < 0.01, ***P < 0.001.

Fig. S4 Elevated activated markers and decreased TCR signaling on TIGIT$^+$ CD8$^+$ T cells

Flow cytometry analysis of the expression of HLA-DR (A), CD38 (B), and CD28 (C) on TIGIT$^-$ vs. TIGIT$^+$ CD8$^+$ T cells from different age groups (n = 27–37 each group). P values were obtained by paired t test or Wilcoxon matched-pairs signed rank test. **P < 0.01, ***P < 0.001.

Fig. S5 TIGIT$^+$ CD8$^+$ T cells from young and middle-aged adults exhibit high susceptibility to apoptosis but no defects in cytokine production

(A–C) Intracellular staining for TNF-α (A), IFN-γ (B), and IL-2 (C) in TIGIT$^-$ and TIGIT$^+$ CD8$^+$ T cells from young and middle-aged adults (21–40 years old for young, n = 16; 41–60 years old for middle-aged, n = 19) upon *in vitro* anti-CD3/anti-CD28 stimulation. (D–E) Percentage of apoptotic cells (7AAD$^-$Annexin V$^+$, D) and expression of CD95 (E) in TIGIT$^-$ and TIGIT$^+$ CD8$^+$ T cells from young and middle-aged adults (21–40 years old for young, n = 21; 41–60 years old for middle-aged, n = 19). P values were obtained by Wilcoxon matched-pairs signed rank test. *P < 0.05, **P < 0.01, ***P < 0.001.

Fig. S6 Dysfunction of Aged TIGIT$^+$ CD8$^+$ T cells can be reversed by transfection of individual TIGIT siRNA

Purified CD8$^+$ T cells from the elderly (n=5-6) were transfected with 2μM indicated siRNA. After cultured *in vitro* for 3 days, expression of TIGIT (A), intracellular cytokine production in response to anti-CD3 and anti-CD28 stimulation (B) and the susceptibility of apoptosis (C) were evaluated by flow cytometry. Representative flow data (left) and plots (right) on CD8$^+$ T cells transfected with 2 individual non-targeting siRNAs vs. 2 individual TIGIT siRNAs are shown. P values were obtained by One-way ANOVA test (TIGIT, TNF-α, Annexin V) or Kruskal-Wallis test (IFN-γ). *P < 0.05, **P < 0.01, ***P < 0.001.

Fig. S7 TIGIT$^+$ CD8$^+$ T cells from the elderly retain the capacity for proliferation and cytotoxicity

Representative flow data (left) and a plot (right) of ki-67 (A), CD107a (B), Granzyme B (C), and perforin (D) expression in TIGIT$^-$ vs. TIGIT$^+$ CD8$^+$ T cells from the elderly (61–80 years old, n = 21). P values were obtained by paired t test or Wilcoxon matched-pairs signed rank test. **P < 0.01, ***P < 0.001.

Fig. S8 CD226 expression on TIGIT$^+$ and TIGIT$^-$ CD8+ T cells from young and middle-aged adults was comparable

Flow cytometry analysis of the expression of CD226 on TIGIT$^-$ vs. TIGIT$^+$ CD8$^+$ T cells from different age groups (n = 27–37 each group). P values were obtained by Wilcoxon matched-pairs signed rank test.

Fig. S1

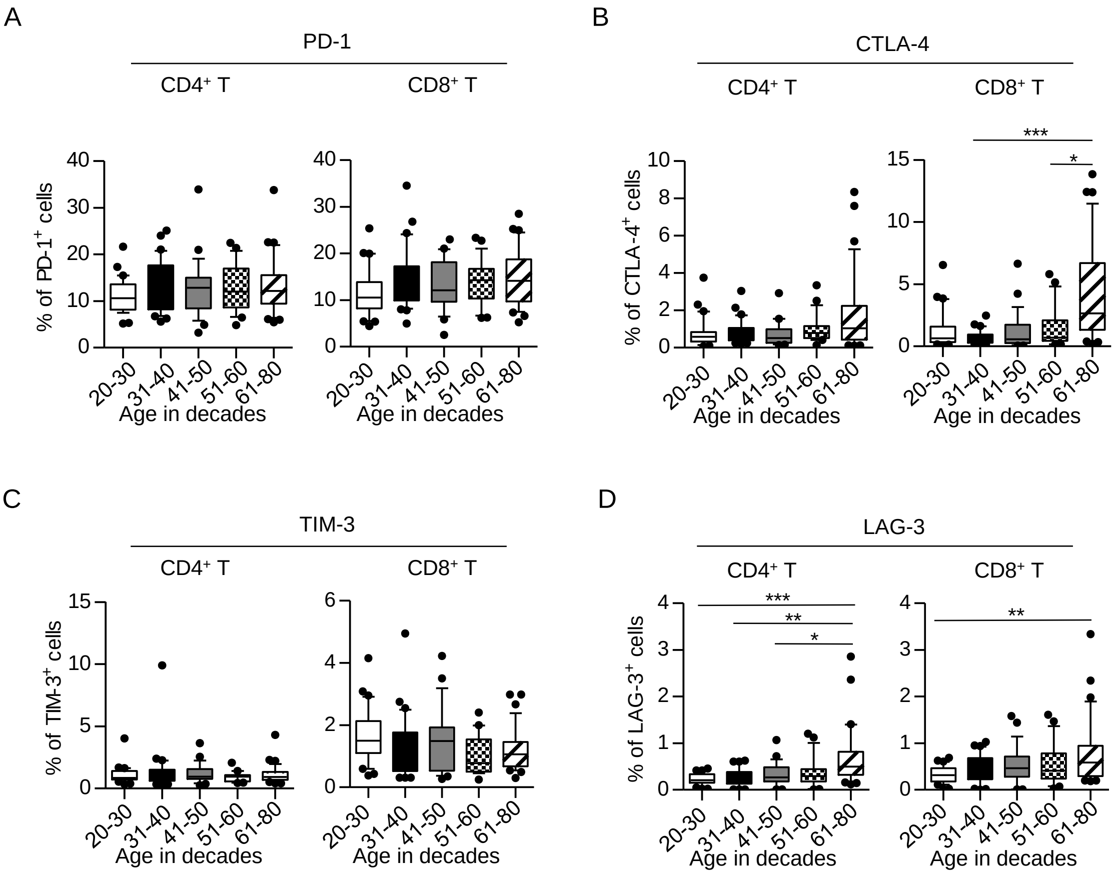
A
PD-1
CD4+ T
CD8+ T
% of PD-1+ cells
Age in decades
B
CTLA-4
CD4+ T
CD8+ T
% of CTLA-4+ cells
Age in decades
C
TIM-3
CD4+ T
CD8+ T
% of TIM-3+ cells
Age in decades
D
LAG-3
CD4+ T
CD8+ T
% of LAG-3+ cells
Age in decades
20-30
31-40
41-50
51-60
61-80

Fig. S2

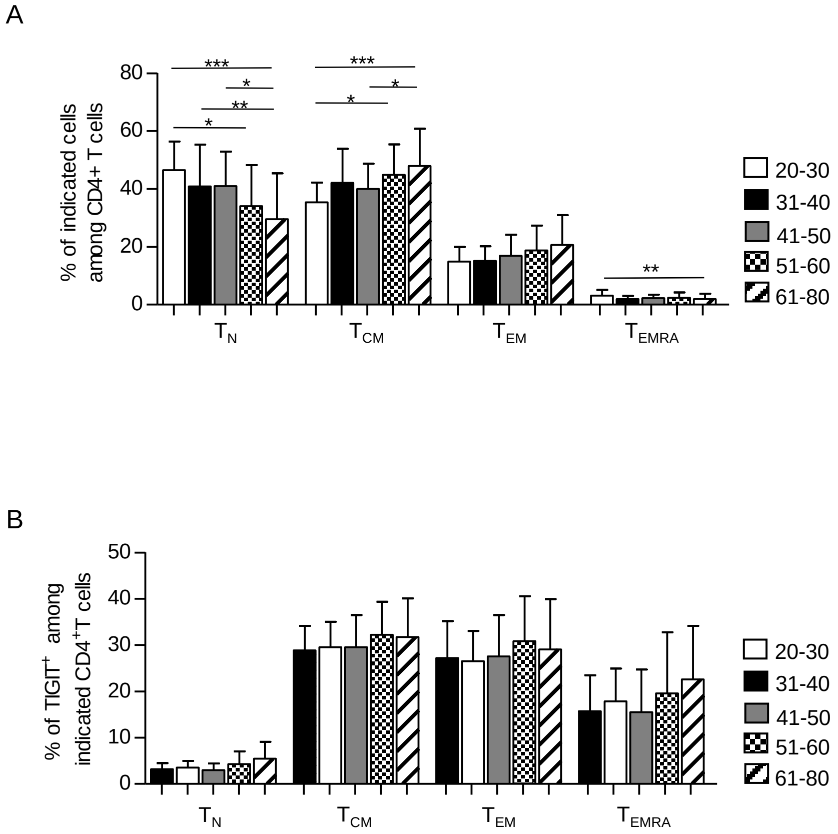
A
80
60
40
20
0
% of indicated cells among CD4+ T cells
***
*
**
*
***
*
*
**
TN
TCM
TEM
TEMRA
20-30
31-40
41-50
51-60
61-80
B
50
40
30
20
10
0
% of TIGIT+ among indicated CD4+T cells
TN
TCM
TEM
TEMRA
20-30
31-40
41-50
51-60
61-80

Fig. S3

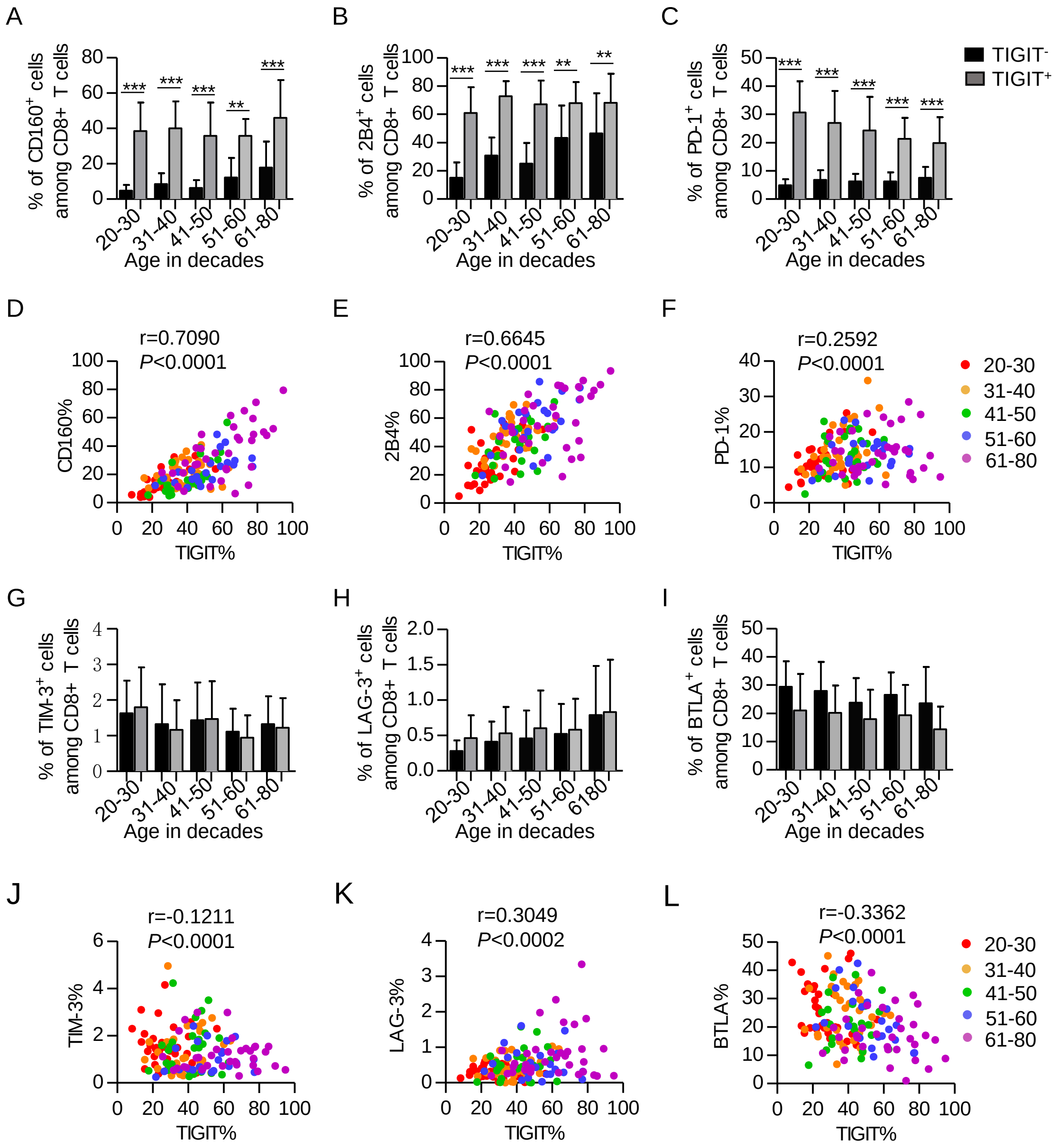

A
B
C
D
E
F
G
H
I
J
K
L
% of CD160+ cells among CD8+ T cells
% of 2B4+ cells among CD8+ T cells
% of PD-1+ cells among CD8+ T cells
TIGIT-
TIGIT+
Age in decades
20-30
31-40
41-50
51-60
61-80
r=0.7090
P<0.0001
r=0.6645
P<0.0001
r=0.2592
P<0.0001
CD160%
2B4%
PD-1%
TIGIT%
% of TIM-3+ cells among CD8+ T cells
% of LAG-3+ cells among CD8+ T cells
% of BTLA+ cells among CD8+ T cells
6180
r=-0.1211
P<0.0001
r=0.3049
P<0.0002
r=-0.3362
P<0.0001
TIM-3%
LAG-3%
BTLA%

Fig. S4

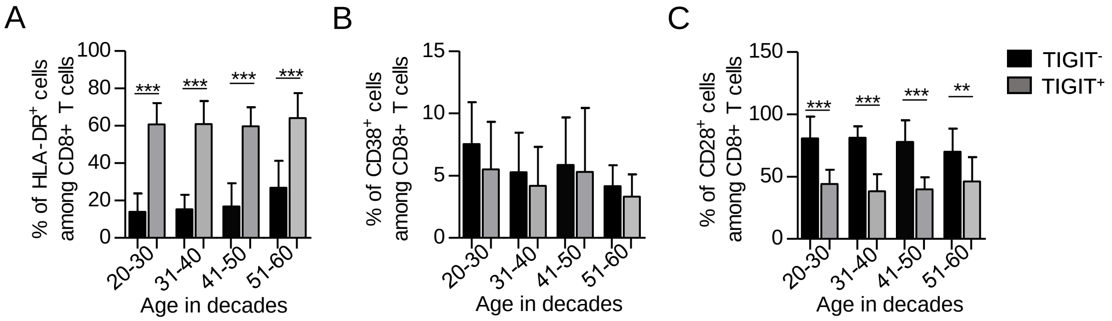
A
% of HLA-DR+ cells among CD8+ T cells
100
80
60
40
20
0
***
***
***
***
20-30
31-40
41-50
51-60
Age in decades
B
% of CD38+ cells among CD8+ T cells
15
10
5
0
20-30
31-40
41-50
51-60
Age in decades
C
% of CD28+ cells among CD8+ T cells
150
100
50
0
***
***
***
**
20-30
31-40
41-50
51-60
Age in decades
TIGIT-
TIGIT+

Fig. S5

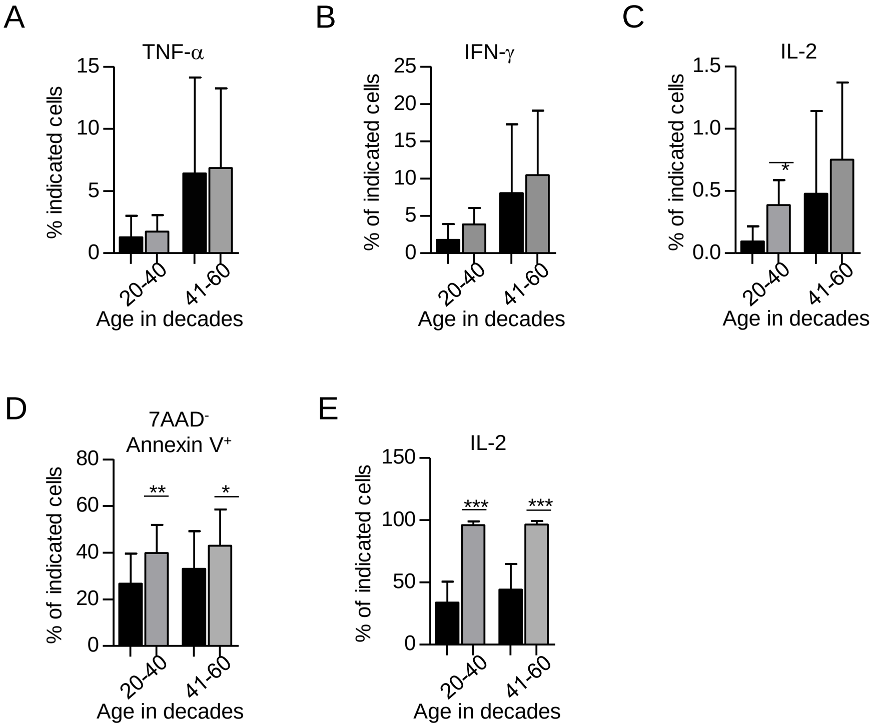
A
TNF-α
% indicated cells
15
10
5
0
20-40
41-60
Age in decades
B
IFN-γ
% of indicated cells
25
20
15
10
5
0
20-40
41-60
Age in decades
C
IL-2
% of indicated cells
1.5
1.0
0.5
0.0
*
20-40
41-60
Age in decades
D
7AAD-
Annexin V+
% of indicated cells
80
60
40
20
0
**
*
20-40
41-60
Age in decades
E
IL-2
% of indicated cells
150
100
50
0
***
***
20-40
41-60
Age in decades

Fig. S6

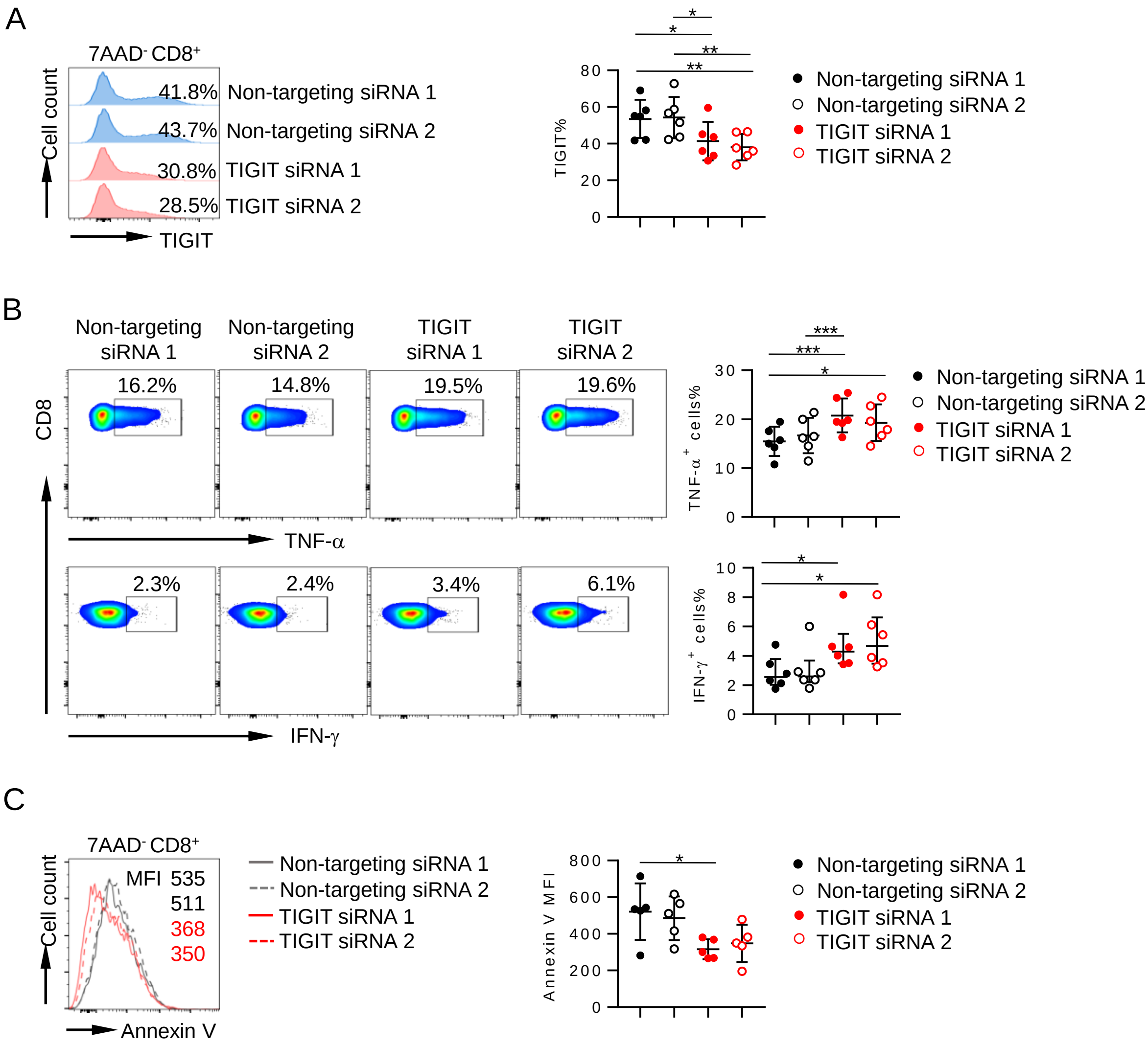

A
7AAD- CD8+
Cell count
41.8% Non-targeting siRNA 1
43.7% Non-targeting siRNA 2
30.8% TIGIT siRNA 1
28.5% TIGIT siRNA 2
TIGIT
TIGIT%
Non-targeting siRNA 1
Non-targeting siRNA 2
TIGIT siRNA 1
TIGIT siRNA 2
B
Non-targeting siRNA 1
Non-targeting siRNA 2
TIGIT siRNA 1
TIGIT siRNA 2
CD8
16.2%
14.8%
19.5%
19.6%
TNF-α
TNF-α+ cells%
2.3%
2.4%
3.4%
6.1%
IFN-γ
IFN-γ+ cells%
C
7AAD- CD8+
Cell count
MFI 535
511
368
350
Annexin V
Annexin V MFI

Fig. S7

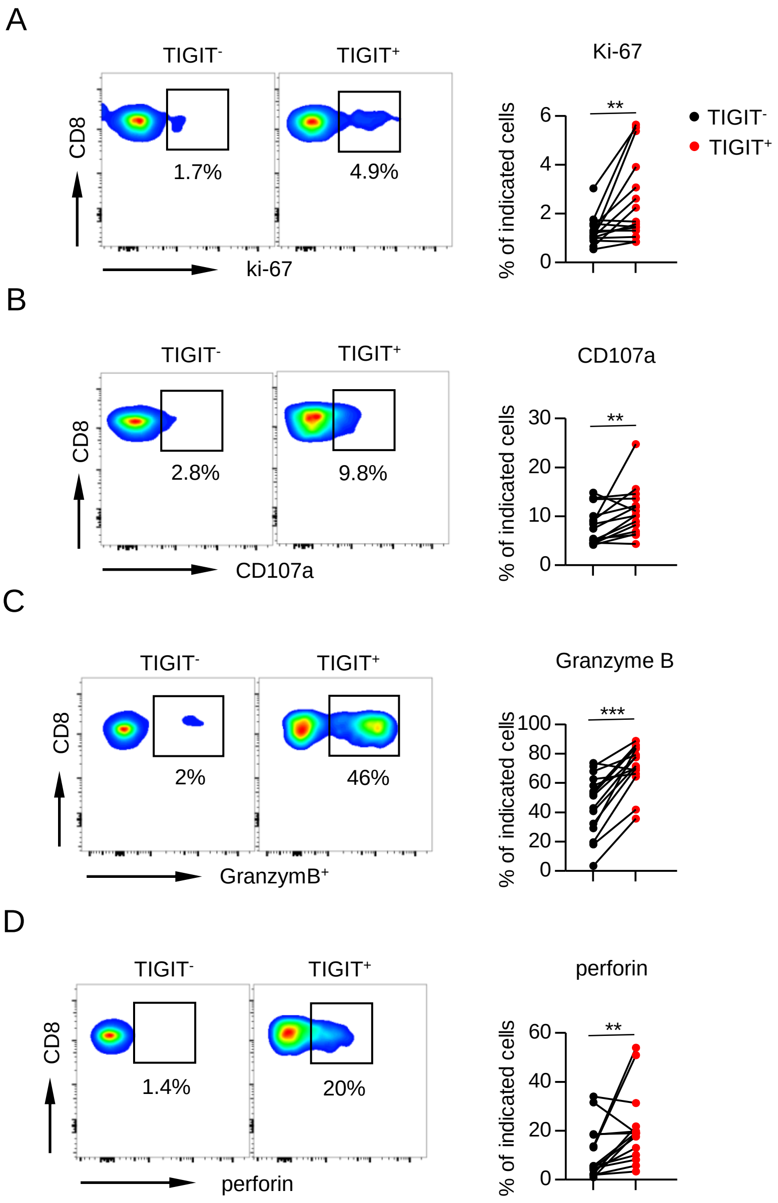
A
TIGIT-
TIGIT+
CD8
1.7%
4.9%
ki-67
Ki-67
**
% of indicated cells
6
4
2
0
TIGIT-
TIGIT+
B
TIGIT-
TIGIT+
CD8
2.8%
9.8%
CD107a
CD107a
**
% of indicated cells
30
20
10
0
C
TIGIT-
TIGIT+
CD8
2%
46%
GranzymB+
Granzyme B
***
% of indicated cells
100
80
60
40
20
0
D
TIGIT-
TIGIT+
CD8
1.4%
20%
perforin
perforin
**
% of indicated cells
60
40
20
0

Fig. S8

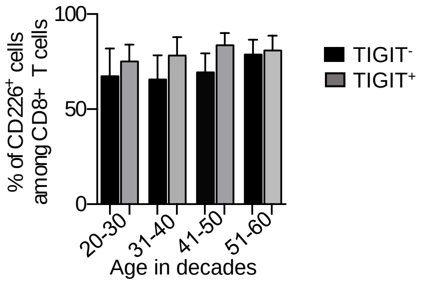
100
50
0
% of CD226+ cells
among CD8+ T cells
20-30
31-40
41-50
51-60
Age in decades
TIGIT-
TIGIT+
