# Supplementary material for: T‐cell Immunoglobulin and ITIM Domain Contributes to CD8+ T‐cell Immunosenescence
Source: Aging Cell. 2018 Jan 19;17(2):e12716. doi: 10.1111/acel.12716 (PMC5847879; doi:10.1111/acel.12716)
Supplement: Supplementary file 2 [file ACEL-17-e12716-s002.docx]

**Supplementary Table 1. List of siRNA sequences used in the study**

| Name | Sequence |
| --- | --- |
| Accell Non-targeting pool siRNA  (including 4 siRNAs)  SMARTpool Accell human TIGIT siRNA  (including 4 siRNAs) | 5’-UGGUUUACAUGUCGACUAA-3’ (D-001910-01)  5’- UGGUUUACAUGUUUUCUGA-3’ (D-001910-02)  5’-UGGUUUACAUGUUUUCCUA -3’ (D-001910-03)  5’- UGGUUUACAUGUUGUGUGA-3’ (D-001910-01)  5’-CUAGGGAACUUGUAGGAAA-3’ (A-018488-13)  5’-CUAUCAUCUUACAAUGUCA-3’ (A-018488-14) |
|  | 5’-GUUUUGUGGUUGAUGAUGA-3’ (A-018488-15) |
|  | 5’-UUGUCAUGAAAAGGUAUUA-3’ (A-018488-16) |
| non-targeting siRNA 1 | 5’-UGGUUUACAUGUCGACUAA-3’ (D-001910-01) |
| non-targeting siRNA 2 | 5’- UGGUUUACAUGUUUUCUGA-3’ (D-001910-02) |
| TIGIT siRNA 1  TIGIT siRNA 2 | 5’-CUAGGGAACUUGUAGGAAA-3’ (A-018488-13)  5’-CUAUCAUCUUACAAUGUCA-3’ (A-018488-14) |
